# The effects of dual modification on functional, microstructural, and thermal properties of tapioca starch

**DOI:** 10.1002/fsn3.2506

**Published:** 2021-07-30

**Authors:** Neda Javadian, Abdorreza Mohammadi Nafchi, Marzieh Bolandi

**Affiliations:** ^1^ Department of Food Science and Technology Islamic Azad University Damghan Iran; ^2^ Food Technology Division School of Industrial Technology Universiti Sains Malaysia Penang Malaysia

**Keywords:** acid hydrolysis, hydroxypropylation, pasting properties, Tapioca starch

## Abstract

The aim of this study was to investigate the effects of dual modification on the functional, microstructural, and thermal properties of tapioca starch. Tapioca starch was first hydrolyzed by 0.14 M HCl for 0, 6, 12, 18, and 24 hr and then hydroxypropylated by adding 0%, 10%, 20%, and 30% (v/w) propylene oxide. The degree of hydroxypropylation, solubility, water absorption, rheological, thermal, and microstructure characterization of dually modified tapioca starch was determined. Hydroxypropylation did not cause any considerable changes in the starch granular size and shape of tapioca starch. Acid hydrolysis disrupts the starch granules, and the starches with smaller sizes were produced. The degree of molar substitution (DS) of dual modified starches ranged from 0.118 to 0.270. The dual modified starches significantly had higher solubility than native starch (*p* < .05). Hydrolysis of starches with acid decreases swelling power while hydroxypropylation increases the swelling power. The results also showed lower gelatinization (To, Tp, Tc, and ΔH) and pasting parameters (the peak and final viscosity, peak time, and pasting temperature) for the dual modified starches than other treatments. In summary, this study showed that dually modified tapioca starch has potential application in dip molding and coating.

## Graphical abstract

- This study aimed to investigate the effects of dual modification on the functional properties of tapioca starch.
- Acid hydrolysis disrupts the starch granules, and the starches with smaller sizes were produced.
- This study showed that dually modified tapioca starch has potential application in dip molding and coating.

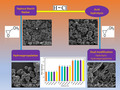

## 1 INTRODUCTION

Starch which is a stored food source in different plants is a homopolysaccharide, and it comprises glucose units that are linked together by glycoside bonds. Starch granules are different in shape, size, and structure (Haq et al., 2019; Ma et al., 2020). Since starches are cheap, biodegradable, renewable, nontoxic, and available, they are widely used in food and nonfood applications (Jafarzadeh et al., 2021). There are several sources of starch, including legumes, cereals, green fruits, tubers, and roots (Ashogbon, 2020; Nafchi & Alias, 2013). Tapioca starch is extracted from cassava roots and is easily available all over the world. Cassava belongs to *Euphoriaceae* family and is widespread in Africa, America, and Asia (Charoenthai et al., 2018; Othman et al., 2019). Cassava global production in 2018 reached 253.4 million tonnes (Prompiputtanapon et al., 2020). Since cassava has few requirements for its growth, it can be easily cultivated (Chuang et al., 2017). Tapioca starch has a low price, high viscosity, bland flavor, clear paste appearance, and low pasting temperature than other starches. Therefore, this starch has wide applications in the food industry (Sheng et al., 2018; Tamimi et al., 2021).

Since native starches have some undesirable properties such as retrogradation, syneresis, and low water holding capacity (WHC), they face limitations in industrial applications (Azaripour & Abbasi, 2020; Russ et al., 2016). Due to the high number of free hydroxyl groups on the structure of starch, these compounds can be easily modified by physical, chemical, biotechnological, or enzymatic methods as well as their combination (Ashogbon, 2020). These modifications are done to improve the texture, adhesion, gel clarity, film making, cooking properties, transparency of gel, freeze‐thaw stability, reduction of retrogradation, the tendency to gel, and syneresis (Mehfooz et al., 2019; Zhang et al., 2021).

Chemical modification of starch structure includes cross‐linking, acid hydrolysis, oxidation, acetylation, grafting, esterification, and dual modification (Haq et al., 2019), which are most used in industry due to easier control (Araghi et al., 2015; Li et al., 2018). Chemical modification of starches can be performed by a single method or a combination of different modification methods. The different properties of the chemically modified starches depend on their botanical source, the nature and number of substitutions, and reaction conditions (Chen et al., 2018).

One of the most practical chemical methods to modify the starches is acid hydrolysis, which improves the different properties of starch, such as improving the resistance to retrogradation, increases water solubility, modification of gelatinization temperatures, and increases hydroxyl groups. This method is often used in the dual modification of starch (Basilio‐Cortés et al., 2019). This method is done by the controlled addition of an acid to an aqueous suspension of starch at a temperature in the normal temperature range slightly below the gelatinization temperature for a certain period (Hoover, 2000). In industry, acid hydrolysis is used to manufacture resistant starches and gelling agents (Aparicio‐Saguilán et al., 2007). Hydroxypropylation (HP), which is a chemical modification method, reduces the hydroxyl group's number in the structure of starch and weakens the hydrogen bonds between the granules. Increasing the degree of hydroxypropylation can increase the freeze‐thaw stability, hydrophilicity, concentration, and clarity of paste (Fouladi & Mohammadi Nafchi, 2014; Park & Kim, 2020).

Today, dual modification of starch structure has attracted the most attention because studies have shown that often the use of single modification methods is not complete and effective to improve the application and processing properties of native starches (Fakharian et al., 2015; Li et al., 2018; Oladzadabbasabadi et al., 2017). Atichokudomchai and Varavinit (2003) investigated the effect of dual modification of tapioca starch by acid hydrolysis and cross‐linking on pharmaceutical tablets and observed that the tablets prepared by dual modified starch were better than those prepared by acid‐modified starch. The researchers also found that the use of dual modification (hydroxypropylation and cross‐linking) improved the properties of taro starch better than using each of these modification methods alone (Hazarika & Sit, 2016).

Due to the confirmation of the effect of chemical modifications on improving the characteristics of starches, this study was conducted to investigate the effect of dual modification of tapioca starch by acid hydrolysis‐hydroxypropylation on its physicochemical, rheological, and thermal properties.

## 2 MATERIALS AND METHODS

### 2.1 Materials

Tapioca starch (12.50% moisture, 0.23% fiber, 0.14% fat, 0.53% ash, 0.12% protein, 21% amylose, and 79% amylopectin) was purchased from SIM Supply Company Sdn. Bhd. (Pulau Penang, Malaysia). All chemicals used in this study were of analytical grade.

### 2.2 Dual modification of tapioca starch by acid hydrolysis‐hydroxypropylation

In order to dual modification of tapioca starch, acid hydrolysis (AH) was performed first, followed by hydroxypropylation (HP). For acid hydrolysis of tapioca starch, about 40% of starch solution was prepared by adding 400 g of starch to a solution of 0.14 N hydrochloric acid at 50°C, and the final weight was 1,000 *g*. The resulting suspension was placed at 50°C for different periods of time (6, 12, 18, and 24 hr) to prepare starch with different molecular weights. The resulting suspension was stirred in a rotary incubator (Jeio Tech SI‐. 600R, South Korean) at 200 rpm to prevent the formation of sediment. After certain periods of time (6, 12, 18, and 24 hr), the starch solutions were neutralized with sodium hydroxide (1% NaOH) until their pH reached 5.5. The samples were washed with distilled water (twice their volume), and then, they were filtered through filter paper (Whatman No. 4). The starch samples were placed in an oven (Memmert, Germany) at 40°C overnight to dry completely (Abdorreza et al., 2012).

To prepare hydroxypropylated tapioca starch, 20% w/v sodium sulfate was added to the 20% w/v hydrolyzed starch solution and stirred. Using 5% sodium hydroxide, the pH of the solution was adjusted above 10.5. Propylene oxide was added as an etherifying agent in different proportions (10%, 20%, and 30% (based on the dry weight of starch)). The samples were then capped and stirred at 20–25°C for 30 min. The resulting solution was then stirred in a rotary incubator at 200 rpm to prevent the formation of sediment. After that, using 10% hydrochloric acid, the pH of the suspension was reduced to 5.5. Samples were immediately washed with distilled water until their sulfate content was negative. By the time the moisture content reached about 10%, the samples were dried in an oven at 40°C. After grinding the starch samples, they were passed through a sieve with a size of 250 µm (Aminian et al., 2013).

### 2.3 Investigation of the granular structure of starches

Scanning electron microscopy (SEM) (Leica Cambridge, England) was used to determining the appearance of starch granules. First, a very small amount of the sample (about 1 g) was glued to a circular metal base made of aluminum, and the surface of the sample was covered with a layer of gold by passing the sputter coater device through a stream of argon gas. The samples were then examined under a microscope with an electric potential of 15 kV (Majzoobi et al., 2012).

### 2.4 Determination of molar substitution (MS)

In order to estimation of molar substitution, 100 mg of the modified sample was poured into a 100‐ml volumetric flask, and 25 ml of 0.5 M sulfuric acid was added. The resulting mixture was placed in a water bath until the color of the solution was light. The solution was immediately cooled and then reached 100 ml with distilled water. 1 mg of the solution was pipetted into a 25‐ml graduated test tube. The test tube was placed in hot water and then transferred to an ice bath. 0.6 ml of ninhydrin solution (3% of ninhydrin in 5% Na_2_S_2_O_5_) was added to the tube and stirred well. It was then placed in a water bath at 25°C for 1 hr. Finally, with concentrated sulphuric acid, it reached a volume of 25 ml. The resulting solution was transferred to a spectrophotometer (Shimadzu, Japan) cell, and its absorbance was read at 590 nm. MS of starch was calculated as follow:

$$\text{MS}\;=\frac{162W}{100‐(M‐1)W}$$

where *w* is the equivalent hydroxypropyl in 100 g of starch, and *M* is the molecular weight of C_3_H_6_O (Fouladi & Mohammadi Nafchi, 2014).

### 2.5 Measurement of solubility and water uptake of starches

In order to measure the solubility of starch in water, 1 g of sample was weighed and poured into a 50 ml plastic centrifuge tube. Then, 30 ml of distilled water was added to the tubes, and tubes were kept in a Bain‐marie at 95°C for 30 min with stirring. The sample temperature was brought to room temperature by cold water for 5 min and centrifuged (Hettich Mikro R220, Germany) at 700 *g* for 15 min. The supernatant clear liquid was carefully transferred to a container of specified weight and dried at 120°C for 4 hr. After drying, the samples were weighed, and the water solubility was calculated using the following equation (Abdorreza et al., 2012):

$$\text{Water}\;\text{solubility}\left(\%\right)=\frac{\text{dried}\;\text{supernatant}\;\text{weight}}{\text{dry}\;\text{starch}\;\text{weight}}\times 100.$$

To determine the water uptake of starches, the centrifuge tubes used in the solubility test were weighed along with the sediments deposited in them after the supernatant was removed, and the amount of water uptake was calculated using the following equation (Abdorreza et al., 2012):

$$\text{Water}\;\text{uptake}\left(\%\right)=\frac{\text{sediment}\;\text{weight}\;\text{inside}\;\text{the}\;\text{centrifuge}\;\text{tube}}{\text{dry}\;\text{starch}\;\text{weight}}\times 100.$$

### 2.6 Investigation of starch pasting properties

Changes in the viscosity of aqueous solutions of starch during heating were investigated by a rapid viscosity analyzer (RVA) (Newport Scientific, Warriewood, Australia). 4 g of starch sample was added to 25 g of distilled water. The desired speed was 960 rpm in the beginning and 160 rpm at the resting stage of the sample. In this experiment, the starch was first kept at 50°C for 1 min and then rapidly increased to 95°C (at a speed of 14°C per minute) and remained at this temperature for about 3.3 min. It was then cooled to 50°C for 4 min and finally kept at this temperature for 2 min. The pasting properties studied in this study included pasting temperature (°C), time to reach peak viscosity (min), peak viscosity (cP), and final viscosity (cP) (Karim et al., 2008).

### 2.7 Investigation of thermal properties of starches

Thermal properties of starch samples, including onset temperature (To), peak temperature (Tp), ending temperature (Te), and gelatinization enthalpy (ΔH), were determined using a differential scanning calorimeter (DSC) (DSC‐Q100, TA Instruments, New Castle, DE, USA). For this purpose, the suspension of starch in distilled water in a ratio of 3:1 (w/w) was weighed in a high‐pressure resistance steel container for the device and kept at room temperature for one day to reach equilibrium. Then, the container containing the sample was placed inside the DSC device, and while heating the sample from 10 to 115°C, the sample was scanned. An empty container of the device was used as a control. The thermal properties of starch samples were calculated and reported from the DSC curves (Majzoobi & Beparva, 2013).

### 2.8 Statistical analysis

Analytical data from tests were analyzed by using one‐way analysis of variance (ANOVA) and Duncan multiply range test to identify the significant difference between samples at *p* < .05 using SPSS software version 22.0.

## 3 RESULTS AND DISCUSSION

### 3.1 Effects of single and dual modification on granular structure tapioca starch

**FIGURE 1:**
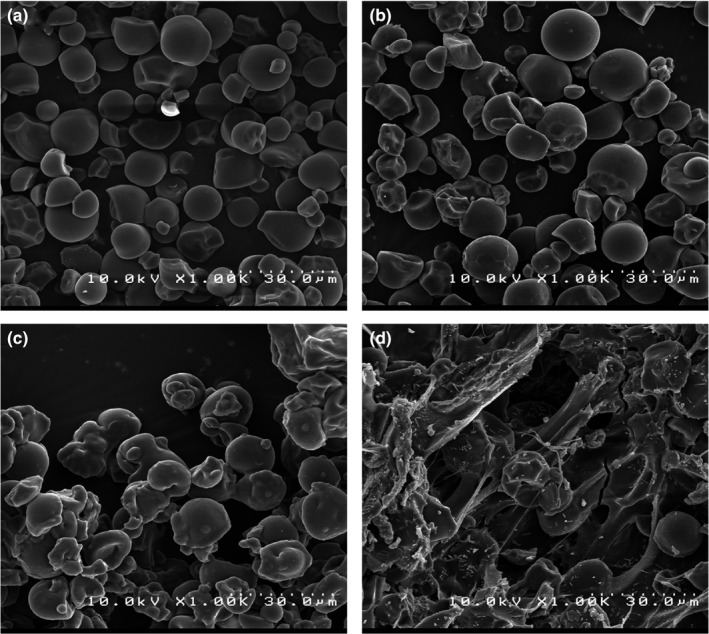
SEM images of native and modified tapioca starch. (a) native starch, (b) acid hydrolyzed starch (at 24 hr hydrolysis), (c) hydroxypropylated starch (30% propylene oxide), (d) dually modified starch (30% propylene oxide and 24 hr hydrolysis)

Scanning electron microscopy images of native tapioca starch and starch samples modified by acid hydrolysis, hydroxypropylation, and dual modification are shown in Figure 1. The native tapioca granules had a spherical shape with a size range of 10–15 µm. The image of hydroxypropylated starch was like that of native starch, while acid hydrolyzed starch came in different shapes and sizes. Dual modification of tapioca starch also caused significant changes in the granules so that in this starch sample, surface roughness was observed, and the granules became irregular. Acidic modification of the starch structure first hydrolyzes the amorphous regions and then attacks the crystalline regions, and both starch amylose and amylopectin are similarly hydrolyzed and reduced to smaller molecular sizes (Wang & Wang, 2001).

Saberi et al. (2013) similarly showed that hydroxypropylation of oat starch did not significantly change the shape and size of starch granules. Tehkhunmag et al. (2008) also observed that hydroxypropylated tapioca starch had SEM images almost similar to native starch, but the dual modification of starch by carboxymethyl/hydroxypropyl caused a significant change in the granular structure of the starch. Mehfooz et al. (2020) stated that the modification of barley starch by dual modification of hydroxypropylation and cross‐linking caused the roughness and grooves formed on the surface of starch granules. Cabrera‐Canales et al. (2020) demonstrated that the granules of achira starch modified by acid hydrolysis were eroded, while the succination caused cavities on the granular structure.

### 3.2 Molar substitution of hydroxypropyl groups (MS)

**FIGURE 2:**
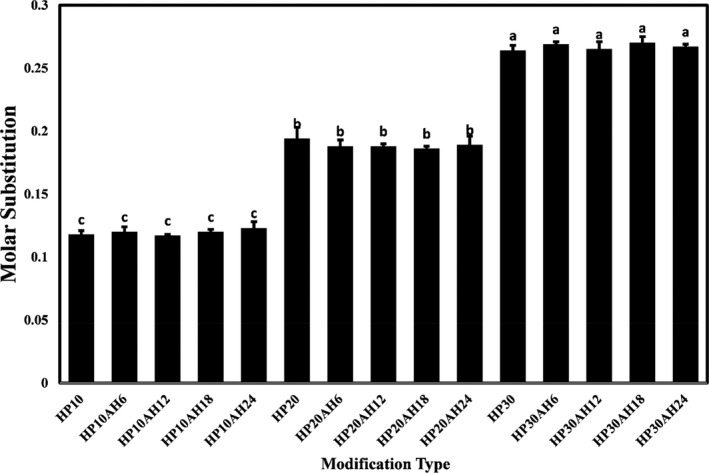
Hydroxypropyl substitution of native and acid hydrolyzed tapioca starches. Bars represents of substitution mean ± *SD* (*n* = 3) of hydroxypropyl group. Different letters show significant difference at 5% level of probability. The numbers after “HP” represent the propylene oxide ratio (%), and the numbers after “AH” represent the time (hours) of acid hydrolysis

In this study, the reaction efficiency of hydroxypropylation and acid hydrolysis was investigated by the degree of molar substitution of hydroxyl groups inside the starch granules. MS involves measuring the average number of hydroxyl groups per unit of anhydroglucose produced by substitution groups (Karim et al., 2008). The MS amount of modified starch should not exceed 3. Since starches from different sources have different structural and functional characteristics, the degree of hydroxyl group substitution in them differs from each other (Fu et al., 2019). MS values of native and modified tapioca starches are shown in Figure 2. MS values of starch samples ranged from 0.117 to 0.270. As the results demonstrated, increasing the ratio of etherifying agent (propylene oxide) from 10% to 30% significantly increased the molar substitution of tapioca starch hydroxyl group (*p* < .05). However, in each of the constant ratios of propylene oxide, increasing the time of acid hydrolysis did not show a significant effect on the MS value of the modified starch samples.

Jerachaimongkol et al. (2006) also reported that an increase in etherifying agents led to an increase in cassava starch MS. Similar results were observed by other researchers on maize starches (Chen et al., 2021). Rutkaitė et al. (2016) also observed that in dual modified potato starches by adding propylene oxide (HP) and octenylsuccinic anhydride (OSA), the increase in OSA had no significant effect on the molar substitution value, and only HP was effective on its amount. Maulani et al. (2013) achieved similar results in investigating the effect of increasing propylene oxide levels on the substitution degree of Arrowroot starch.

### 3.3 Water solubility of modified tapioca starch

The starch solubility values of native and modified tapioca starch samples are given in Table 1 and show that the lowest solubility was observed in native starch (52.99%), and modification of starches resulted in a significant increase in the solubility of starch samples (*p* < .05). Increasing the oxidizing agent ratio from 10% to 30% and hydrolysis time from 6 to 24 hr also caused a significant increase in water solubility of starches (*p* < .05). The highest solubility was obtained in the dually modified starch at the highest oxidizing agent ratio and the highest acid hydrolysis time (HP30AH24) (99.49%). According to the results of the microscopic structure of tapioca starches, the increase in solubility of samples due to acid hydrolysis can be interpreted. As the SEM images showed, acid hydrolysis to some extent caused the degradation of starch granules, which in turn increased the amount of amylose out of the granules, resulting in increased solubility in water. Hydroxypropylation also increased the solubility of tapioca starch. This increase in the solubility of starches is associated with placing hydroxypropyl groups with –OH groups in the structure of starch. These hydroxyl groups are hydrophilic and increase access to water molecules (Rutenberg & Solarek, 1984). Tehkhunmag et al. (2008) also observed an increase in the solubility of tapioca starch samples due to the hydroxypropylation process. Biduski et al. (2017) showed that dual modification of sorghum starch by acid hydrolysis and oxidation resulted in a significant increase in water solubility.

**TABLE 1:** Water solubility and water absorption of tapioca starches (native and modified)

Starches	Water solubility (%)	Water absorption (%)
Native	52.99 ± 0.65 l	17.23 ± 0.56 d
AH6	64.86 ± 0.52 k	8.89 ± 0.52 h
AH12	70.14 ± 0.53 j	5.53 ± 0.42 k
AH18	75.23 ± 0.75 i	3.11 ± 0.71 m
AH24	77.51 ± 0.51 h	0.94 ± 0.25 p
HP10	75.75 ± 0.59 i	18.59 ± 0.42 c
HP10AH6	84.70 ± 0.44 f	10.15 ± 0.48 g
HP10AH12	88.64 ± 0.52 e	6.59 ± 0.45 j
HP10AH18	92.47 ± 0.58 d	4.35 ± 0.49 l
HP10AH24	95.10 ± 0.73 c	1.54 ± 0.31 op
HP20	81.36 ± 0.80 g	19.56 ± 0.48 b
HP20AH6	88.99 ± 0.74 e	11.23 ± 0.47 f
HP20AH12	92.22 ± 0.40 d	7.47 ± 0.45 ij
HP20AH18	94.67 ± 0.53 c	5.15 ± 0.32 kl
HP20AH24	98.23 ± 0.52 b	1.89 ± 0.31 no
HP30	88.09 ± 0.59 e	20.58 ± 0.32 a
HP30AH6	62.67 ± 0.38 d	12.14 ± 0.42 e
HP30AH12	95.17 ± 0.49 c	7.90 ± 0.39 i
HP30AH18	97.81 ± 0.73 b	5.44 ± 0.28 k
HP30AH24	99.49 ± 0.42 a	2.38 ± 0.20 mn

Note

The values are mean ± *SD* (*n* = 3). Different letters show significant difference at 5% level of probability between values in same columns. The numbers after “HP” represent the propylene oxide ratio (%), and the numbers after “AH” represent the time (hours) of acid hydrolysis.

### 3.4 Water uptake of modified tapioca starch

The amount or strength of water absorption indicates the hydration capacity and the rate of reaction between the starch chains in the amorphous regions and the crystalline regions. Table 1 shows the water uptake percentage values of native and modified tapioca starch samples. As the results demonstrated, the hydroxypropylation process led to a significant increase in water uptake by starch granules and increasing the oxidizing agent from 10% to 30% also significantly increased water uptake percentage (*p* < .05). However, acid hydrolysis of starch samples showed an opposite effect on water uptake, so that increasing the time of hydrolysis significantly reduced the amount of water uptake of starch samples (*p* < .05). The lowest water uptake percentage in modified starch was obtained at the highest hydrolysis time (AH6) (0.94%), and the highest amount was for HP30 (20.58%). Reduction of water uptake of starch granules by acid hydrolysis can be attributed to partial degradation of granule structure by acid treatment. Because due to the destruction of starch granules, their water holding capacity is reduced, and as a result, the water uptake is reduced. Increasing the water uptake of hydroxypropylated starches is due to the fact that due to the entry of hydroxypropyl groups into the starch chains, intramolecular and intermolecular hydrogen bonds are destroyed, and the water uptake of granules increases. Higher water uptake in hydroxypropylated starches is also related to the hydrophilic nature of hydroxypropyl groups (Pal et al., 2002).

Sriburi et al. (1999) found that the presence of ascorbic acid on starch caused a series of changes, including a decrease in the swelling strength of granules, followed by a decrease in its water uptake. However, Okunlola and Akingbala (2013) demonstrated that acid hydrolysis of Chinese sweet potato starch increased the water uptake and solubility of starch granules. Mehboob et al. (2021) also found an increase in the water holding capacity of sorghum starch due to the introduction of hydroxypropyl groups into the starch structure. Increased water uptake percentage of different starches due to the hydroxypropylation process has been reported by different researchers (Karim et al., 2008; Park & Kim, 2020; Tehkhunmag et al., 2008).

### 3.5 Effects of modification on pasting properties of tapioca starch

The pasting properties of native and modified tapioca starch samples are compared in Table 2. The highest pasting temperature was observed in native tapioca starch (71.04°C), and starch modification by acid hydrolysis and hydroxypropylation resulted in a significant decrease in this temperature (*p* < .05). Increasing the oxidant agent and hydrolysis time also led to a significant reduction in the pasting temperature, so that the lowest pasting temperature was obtained in HP30AH24 (49.11°C). In terms of peak viscosity time, native starch had the longest time (5.70 min), and modified starch samples took less time to reach peak viscosity (*p* < .05). With increasing propylene oxide ratio as well as hydrolysis time, the time to reach peak viscosity decreased (*p* < .05). The lowest time to reach peak viscosity was observed in HP30AH24 (2.00 min). Native tapioca starch also had the highest peak viscosity (2,160.2 cP) and final viscosity (1,989.4 cP) and was reduced by dual modifying the structure of the starch (*p* < .05). The effect of hydroxypropylation process on reducing the peak viscosity was greater than the acid hydrolysis so that in hydroxypropylated treatments alone, the peak viscosity was significantly lower than acid hydrolyzed treatments (*p* < .05). The lowest peak and final viscosities were obtained in HP30AH24 modified starch (2,004.8 and 1,912.5 cP, respectively). Dual modified starches showed lower final viscosity than hydrolyzed and hydroxypropylated starches.

**TABLE 2:** Pasting properties of tapioca starches (natural and modified)

Starches	Pasting temperature (°C)	Time to reach peak viscosity (min)	Peak viscosity (cP)	Final viscosity (cP)
NTS	71.04 ± 0.31 a	5.70 ± 0.04 a	2,160.2 ± 7.0 a	1,989.4 ± 2.0 a
AH6	65.70 ± 0.26 b	5.42 ± 0.12 b	2,145.9 ± 4.8 b	1,985.4 ± 1.4 b
AH12	65.27 ± 0.11 c	5.23 ± 0.08 c	2,143.0 ± 2.8 b	1,979.9 ± 2.5 c
AH18	64.96 ± 0.24 c	5.09 ± 0.06 cd	2,140.6 ± 3.9 b	1,974.4 ± 2.2 d
AH24	64.38 ± 0.21 d	4.93 ± 0.11 d	2,139.2 ± 1.6 b	1,968.5 ± 3.4 e
HP10	57.80 ± 0.36 e	3.85 ± 0.14 e	2,125.8 ± 1.3 c	1,965.3 ± 1.9 ef
HP10AH6	54.48 ± 0.25 g	3.63 ± 0.07 f	2,109.1 ± 10.5 d	1,960.8 ± 2.6 f
HP10AH12	53.95 ± 0.24 h	3.49 ± 0.10 fg	2,083.1 ± 18.3 e	1,957.1 ± 1.1 g
HP10AH18	53.52 ± 0.13 ij	3.35 ± 0.10 gh	2,047.9 ± 13.2 fg	1,955.1 ± 1.2 gh
HP10AH24	53.27 ± 0.14 ij	3.20 ± 0.06 hi	2,037.5 ± 7.2 gh	1,952.7 ± 1.5 hi
HP20	55.42 ± 0.33 f	3.13 ± 0.23 i	2,091.6 ± 2.7 e	1,955.4 ± 2.0 gh
HP20AH6	53.68 ± 0.28 hi	2.75 ± 0.12 j	2,056.8 ± 5.0 f	1,950.9 ± 2.4 i
HP20AH12	53.46 ± 0.19 ij	2.51 ± 0.10 kl	2,045.9 ± 4.5 fg	1,946.1 ± 2.3 j
HP20AH18	53.16 ± 0.15 jk	2.26 ± 0.12 mn	2,038.0 ± 3.1 gh	1,942.3 ± 1.2 k
HP20AH24	52.84 ± 0.18 kl	2.17 ± 0.09 n	2,030.6 ± 5.4 h	1,939.3 ± 2.0 k
HP30	52.60 ± 0.30 l	2.70 ± 0.13 jk	2,044.6 ± 2.5 g	1,929.6 ± 2.4 l
HP30AH6	50.44 ± 0.18 m	2.51 ± 0.09 kl	2,019.8 ± 4.2 i	1,924.0 ± 2.3 m
HP30AH12	49.92 ± 0.25 n	2.35 ± 0.11 lm	2,013.1 ± 3.3 ij	1,920.2 ± 1.5 n
HP30AH18	49.42 ± 0.23 o	2.14 ± 0.09 n	2,009.5 ± 4.3 ij	1,917.0 ± 2.4 n
HP30AH24	49.11 ± 0.19 o	2.00 ± 0.03 o	2,004.8 ± 6.3 j	1,912.5 ± 2.2 o

Note

The values are mean ± *SE* (*n* = 3). Different letters show significant differences at 5% level of probability between values in the same columns. The numbers after “HP” represent the propylene oxide ratio (%), and the numbers after “AH” represent the time (hours) of acid hydrolysis.

Peak viscosity is defined as the equilibrium point between swelling and solubility (increases viscosity) and the fracture and placement of polymer chains (reduces viscosity). Due to hydroxypropylation of starch, water absorption by granules is facilitated because the hydroxypropyl subgroup is a hydrophilic group and causes more water and starch connections. Increased water uptake and hydrophilic strength after hydroxypropylation increase the amylose release rate mainly in amorphous starch regions, and as a result, the solubility increases (Kaur et al., 2004; Lawal, 2009). Hence, reaching the peak viscosity takes place at a lower temperature and time. After reaching the peak viscosity, with increasing temperature and passing the gelatinization temperature, the phenomenon of pasting occurs, which is accompanied by complete destruction of the granular structure and the release of water from the granules to the surrounding environment, resulting in decreased viscosity. During cooling, the starch molecules, especially amylose, are reorganized, and water is trapped inside the starch chains. As a result, viscosity increases due to gel formation, known as the final viscosity (Perera & Hoover, 1999). The results of this study showed that the use of acid hydrolysis reduced the peak and final viscosities and the time to reach the peak viscosity. Smaller molecules often produce less viscosity, so the viscosity reduction was not unexpected due to the reduction in the size of the starch molecules due to acid hydrolysis. According to the findings of Thys et al. (2013), acid hydrolysis reduces the molecular weight of starch and increases free aldehyde groups, thus reducing viscosity and increasing starch solubility. Saberi et al. (2013) similarly showed that hydroxypropylation of wheat and barley starches reduced the peak viscosity, storage time, and pasting temperature of starches. Majzoobi and Beparva (2013) observed that acidic hydrolysis of wheat starch with acetic acid significantly reduced peak and final viscosities and time to reach peak viscosity, which was consistent with the results of the present study. Biduski et al. (2017) also observed that starches modified by acid hydrolysis and oxidation had a lower pasting temperature than natural sorghum starch, indicating starch depolymerization due to acid modification and oxidation.

### 3.6 Effects of modification on thermal properties of tapioca starch

The thermal properties of native and modified tapioca starch samples are presented in Table 3. As can be seen in the table, hydroxypropylated starches had significantly lower To, Tp, and Tc than native and hydrolyzed starches (*p* < .05). Increasing the oxidizing agent ratio from 10% to 30% caused a significant decrease in To, Tp, and Tc (*p* < .05). However, acid hydrolysis and its time did not show a significant effect on gelatinization temperatures. The To, Tp, and Tc values of different tapioca starches in this study were in the range of 53.15–63.26°C, 66.61–75.41°C, and 75.25–82.16°C, respectively.

**TABLE 3:** DSC properties of tapioca starches (natural and modified)

Starches	To (°C)	Tp (°C)	Tc (°C)	ΔH (J/g)
NTS	63.26 ± 0.13 a	75.39 ± 0.29 a	82.16 ± 0.12 a	14.40 ± 0.17 a
AH6	63.23 ± 0.11 a	75.41 ± 0.09 a	82.12 ± 0.10 a	14.37 ± 0.15 a
AH12	63.12 ± 0.09 a	75.30 ± 0.16 a	82.11 ± 0.08 a	14.33 ± 0.12 a
AH18	63.06 ± 0.08 a	75.18 ± 0.17 a	82.02 ± 0.13 a	14.25 ± 0.10 a
AH24	63.06 ± 0.07 a	75.16 ± 0.12 a	81.97 ± 0.18 a	14.18 ± 0.07 a
HP10	59.74 ± 0.09 b	72.89 ± 0.16 b	80.46 ± 0.24 bc	12.18 ± 0.23 b
HP10AH6	59.69 ± 0.13 b	72.82 ± 0.18 b	80.51 ± 0.11 b	12.24 ± 0.12 b
HP10AH12	59.61 ± 0.08 b	72.70 ± 0.09 b	80.40 ± 0.11 bc	12.16 ± 0.10 b
HP10AH18	59.57 ± 0.09 b	72.73 ± 0.07 b	80.29 ± 0.18 bc	11.99 ± 0.14 bc
HP10AH24	59.54 ± 0.12 b	72.62 ± 0.11 b	80.21 ± 0.12 c	11.86 ± 0.14 c
HP20	55.98 ± 0.18 c	70.04 ± 0.14 c	78.03 ± 0.28 d	11.01 ± 0.24 d
HP20AH6	55.98 ± 0.12 c	70.07 ± 0.11 c	78.10 ± 0.08 d	11.00 ± 0.18 d
HP20AH12	55.96 ± 0.09 c	70.03 ± 0.12 c	78.01 ± 0.19 d	10.92 ± 0.19 d
HP20AH18	55.85 ± 0.13 cd	69.94 ± 0.12 c	77.87 ± 0.17 de	10.82 ± 0.18 de
HP20AH24	55.72 ± 0.14 d	69.82 ± 0.13 c	77.71 ± 0.16 e	10.57 ± 0.19 e
HP30	53.17 ± 0.15 e	66.78 ± 0.25 d	75.46 ± 0.11 f	9.03 ± 0.19 f
HP30AH6	53.17 ± 0.08 e	66.89 ± 0.09 de	75.32 ± 0.09 f	8.98 ± 0.17 f
HP30AH12	53.21 ± 0.09 e	66.75 ± 0.14 de	75.30 ± 0.13 f	8.89 ± 0.12 f
HP30AH18	53.15 ± 0.12 e	66.69 ± 0.15 de	75.25 ± 0.13 f	8.82 ± 0.11 f
HP30AH24	53.17 ± 0.10 e	66.61 ± 0.12 e	75.26 ± 0.16 f	8.75 ± 0.06 f

Note

The values are mean ± *SE* (*n* = 3). Different letters show significant differences at 5% level of probability between values in the same columns. To, onset temperature; Tp, peak temperature; Tc, completion temperature; ΔH, enthalpy. The numbers after “HP” represent the propylene oxide ratio (%), and the numbers after “AH” represent the time (hours) of acid hydrolysis.

The results of starch enthalpy also demonstrated that the hydroxypropylation led to a significant reduction in enthalpy, and with increasing the ratio of propylene oxide from 10% to 30%, the enthalpy also decreased significantly (*p* < .05). In general, acid hydrolysis had no significant effect on the enthalpy of tapioca starches. Enthalpy values of different samples of tapioca starch ranged from 8.75 to 14.40 J/g. The enthalpy of gelatinization is a measure of the quality and quantity of crystals and the loss of molecular order within the granules (Cooke & Gidley, 1992). Low enthalpy actually indicates the structural order and less stability of the crystals. The entry of large groups of hydroxypropyl into the polymeric structure of starch destroys the double helix in the amorphous regions, thereby increasing the structural flexibility and decreasing the melting point (Perera et al., 1997). The hydroxypropylation also destroys intramolecular and intermolecular hydrogen bonds, reducing crystallinity in starch granules, increasing water access to starch granules, and reducing enthalpy (Liu et al., 1999; Woggum et al., 2015).

Chen et al. (2021) reported a reduction in gelatinization temperature and enthalpy in dually modified maize starches modified by acid hydrolysis and hydroxypropylation. Lee and Yoo (2011) reported that hydroxypropylated sweet potato starches had lower gelatinization temperatures and enthalpy than native starch. Wang and Shi (2020) found that due to hydroxypropylation of waxy wheat, normal wheat, and waxy maize starches, their gelatinization temperatures and enthalpy significantly decreased. Gunaratne and Corke (2007) also reported that hydroxypropylation reduced the gelatinization temperatures and enthalpy of starch. They attributed this decrease to the fact that excessive amounts of hydrated granules require less force and energy for gelatinization.

## 4 CONCLUSION

Due to the low water solubility and tendency to retrograde, the use of native starches in industrial applications is often difficult. By introducing hydrophilic hydroxypropyl groups into the structure of tapioca starch, the water solubility and water uptake capacity of the resulting modified starch were significantly improved. Modification of tapioca starch in this study also improved the order of molecular structure and reduced the stability of granules. Therefore, it reduced the force and energy required to gelatinize the starch. According to the results obtained in this study, dually modified tapioca starch can be used in dip molding and coating industries.

## CONFLICT OF INTEREST

The authors declare no conflict of interest.

## AUTHOR CONTRIBUTIONS

**Neda Javadian:** Formal analysis (equal); Investigation (equal); Methodology (equal); Resources (equal); Visualization (equal); Writing‐original draft (equal). **Abdorreza Mohammadi Nafchi:** Conceptualization (equal); Data curation (equal); Funding acquisition (equal); Project administration (equal); Supervision (equal); Writing‐review & editing (equal). **Marzieh Bolandi:** Project administration (equal); Supervision (equal); Validation (equal); Writing‐review & editing (equal).

## ETHICAL APPROVAL

This study does not involve any human or animal testing.

## Data Availability

The data that support the findings of this study are available from the corresponding author, upon reasonable request.
